# The Wrong Fix: Why America Doesn’t Need More Medical Schools to Solve the Physician Shortage

**DOI:** 10.7759/cureus.85935

**Published:** 2025-06-13

**Authors:** Shaheen E Lakhan

**Affiliations:** 1 Medical Office, Click Therapeutics, Inc., New York, USA; 2 Department of Neurology, Western University of Health Sciences, Pomona, USA; 3 Department of Neurology, A.T. Still University School of Osteopathic Medicine in Arizona, Mesa, USA; 4 Department of Neurology, Morehouse School of Medicine, Atlanta, USA; 5 Department of Bioscience, Global Neuroscience Initiative Foundation, Miami, USA

**Keywords:** ai-augmented supervision, graduate medical education, healthcare access, healthcare workforce, medical education, medical school expansion, physician shortage, policy reform, residency cap, residency training

## Abstract

The United States is grappling with a physician shortage, but the solution does not lie in simply opening more medical schools. As a physician-scientist and former founding dean of a medical school, I argue that the true bottleneck is not the number of medical school graduates but the insufficient number of residency training positions. Since the Balanced Budget Act of 1997, which froze the number of Medicare-funded residency slots, the United States has seen a steady increase in medical graduates, yet the availability of residency spots has stagnated. This mismatch between undergraduate medical education (UME) expansion and the lack of corresponding growth in graduate medical education (GME) is the key issue. This editorial explores the structural flaws in the current system, particularly the artificial cap on residency slots, and critiques the recent push to open new medical schools without addressing the underlying problem in residency training. Drawing on personal experience and data, I examine the consequences of this mismatch, including the vast number of unmatched graduates, a growing physician shortage, and the inefficient use of educational resources. I propose policy reforms, including lifting the federal GME cap, creating public-private partnerships, leveraging technology for AI-augmented supervision, and adopting hybrid training models to modernize GME. Only by expanding and modernizing residency programs in tandem with UME growth can the U.S. effectively resolve its physician shortage and ensure that the medical graduates of today are trained and ready to meet the healthcare needs of tomorrow.

## Editorial

Introduction

As a physician-scientist and former founding dean of a medical school in the twenty-first century, I have witnessed every stage of the physician pipeline, from the eager aspirants in undergraduate medical education (UME) to the seasoned clinicians honing their craft in graduate medical education (GME) and beyond. Over decades of leadership in medical education, health system administration, and therapeutic innovation, one reality has become inescapably clear: America’s physician shortage is not for lack of medical school graduates but for lack of residency training positions. In other words, we have opened the spigot to produce more medical students, but the bottleneck at the residency stage remains the rate-limiting step. This editorial argues that the long-standing cap on residency slots, originating with the 1997 Balanced Budget Act, is the key structural constraint throttling the U.S. physician workforce [1]. It further contends that the current rush to open new medical schools without commensurate investment in residency training is a misguided strategy that will fail to resolve the shortage. Drawing on personal experience and data, I illustrate the mismatch between UME expansion and stagnation in GME and explore forward-looking solutions, from policy reforms to technology-augmented training models, to expand and modernize residency education in the United States.

More Graduates, Nowhere to Go: The Residency Slot Bottleneck

Since 1952, each spring on Match Day, thousands of freshly minted medical graduates learn whether they will progress into residency programs, the mandatory next step in physician training. In March 2025, 47,208 applicants (including U.S. MD and DO seniors and international graduates) vied for only 37,667 first-year residency positions [2]. A total of 9,541 qualified medical graduates went unmatched, unable to obtain a residency slot, and therefore unable to become licensed physicians [2]. To note, this does not include registrants that simply withdrew (n=2,315) or did not submit a rank-list (n=2,975), often because they were not offered interviews [2]. I still remember counseling a distraught top student from our early medical school class who, despite excellent credentials, failed to match in that bottleneck. Such stories are far from rare. Every year, thousands of graduates are left stranded by the shortfall of GME positions, a tragic waste of talent and ambition that also deprives patients of would-be physicians. While a few unmatched graduates scramble into unfilled positions or reapply the next year, most face the grim reality that without residency, they cannot practice medicine in the United States. All 50 states require at least one year of residency for medical licensure (and most require three years or more). An MD diploma alone is thus no ticket to healing patients; GME is the choke point that determines whether a medical school graduate can actually serve as a physician.

Importantly, this residency gridlock is a policy choice, not an inevitability of training logistics. In fact, the total number of residency slots available in the U.S. has long been artificially constrained by federal funding caps. Meanwhile, the number of medical students graduating has surged in recent years. When all applicants are considered, there are only about 0.82 residency positions per applicant, meaning a significant fraction of doctors-in-training will inevitably go unmatched each year. The bottleneck thus affects not only U.S.-trained graduates but also many foreign-trained physicians eager to help address U.S. healthcare needs. In my role as a health system leader, I have seen communities desperately try to recruit physicians while an untapped pool of medical graduates sits on the sidelines due to insufficient training opportunities. This mismatch between UME and GME is at the heart of the physician workforce shortage.

The 1997 Residency Cap: A Legacy of an Outdated Policy

Why hasn’t the number of residency positions kept up with the number of medical graduates or the obvious needs of a growing and aging population? The answer traces back to a decision made in the late 1990s that is still constraining us today. In 1997, Congress passed the Balanced Budget Act (BBA), a sweeping law intended to trim federal expenditures, and one target was GME funding [1]. At that time, Medicare was (and remains) the single largest sponsor of residency training, reimbursing teaching hospitals for a portion of resident stipends and associated teaching costs [3]. Concerned about rising healthcare costs (and swayed by erroneous forecasts of a physician surplus), lawmakers froze Medicare’s GME support in its tracks. The BBA established hospital-specific caps on the number of residents that Medicare would fund, effectively fixing each teaching hospital’s funded residency slots at the level they had in 1996 [1,3]. In practical terms, if a teaching hospital wanted to expand its residency programs beyond that 1996 baseline, Medicare would not pay a dime for the additional trainees. This drastic measure, accomplished with little fanfare outside policy circles, slammed the brakes on GME growth nationwide.

At the time, remarkably, much of organized medicine supported the cap. In the 1990s, influential groups like the American Medical Association (AMA) and Association of American Medical Colleges (AAMC) had become convinced the U.S. faced an impending “physician oversupply.” In March 1997, just months before the BBA’s passage, a consortium of medical organizations, including the AMA, went so far as to recommend reducing the number of residency positions by 25% (from ~25,000 slots down to 19,000) to stave off a surplus [4]. “The United States is on the verge of a serious oversupply of physicians,” the AMA and others warned at the time. With that mindset, capping residency funding seemed a reasonable cost-saving step to lawmakers, who had cover from these expert groups to impose GME limits. Little did they know how dramatically the landscape would shift in the coming decades.

Fast-forward to today, and what was once thought to be a glut of doctors has proven to be a shortage. The BBA’s residency cap has remained essentially intact for over 25 years, even as our population grew by over 70 million and aged significantly. From 1997 to 2002, the five years immediately after the cap, the total number of residents in training increased by a minuscule 0.1%, essentially flatlining growth [4]. In the two decades since, the number of residency positions has crept upward at an anemic pace, supported only by piecemeal efforts and workaround programs, but never coming close to the demand. Each year, thousands of residency applicants fail to secure a position, a direct consequence of the cap set in 1997 that still limits how many new physicians we train annually. In short, the BBA’s cap on Medicare-funded residency slots, a policy fossil, has become the single largest structural bottleneck in the physician supply line. Even as medical schools graduate more students, the cap constrains the throughput of new practicing doctors.

It is worth noting that there have been modest adjustments to the cap over the years, but these have been too small to make more than a dent. For example, in 1999, Congress allowed limited increases for rural hospitals, and the Affordable Care Act of 2010 redistributed a small number of unused training slots to primary care and rural programs. Most recently, in 2020, Congress authorized funding for 1,000 new Medicare-supported residency positions to be added gradually over 5 years [5]. While welcome, 1,000 slots spread across the nation is a drop in the bucket, barely a 2-3% increase in residency capacity, phased in over half a decade. In contrast, the AAMC projects a U.S. physician shortage of up to 139,000 doctors by 2033 if current trends continue [6]. To put it bluntly, the 1997 cap remains the chokehold on physician production, and Congress’s “baby steps” toward lifting it have been nowhere near commensurate with the need. As one analysis noted, to fully meet future demand, we would need to roughly double the number of new residency positions for several years - an implausible scenario under the status quo. The cap persists as the defining limit on our ability to train the doctors that American patients require.

Opening New Medical Schools: A Misguided Solution if GME Isn’t Addressed

Over the past 15 years, many well-intentioned efforts have focused on expanding the front end of the pipeline, the medical schools, in response to warnings of a doctor shortage. I was part of one such effort when I helped establish a new medical school in the 2010s. The logic seemed straightforward: more medical school seats would eventually equal more practicing physicians. Indeed, since 2006, the AAMC has encouraged a 30% expansion in U.S. medical school enrollment, a goal that has essentially been met. Between 2002 and 2020, enrollment in MD-granting medical schools grew by nearly 35% [7]. This has been achieved through a combination of class size expansion at established schools and the launch of approximately 60 new medical schools in recent decades [8]. From the perspective of UME, we are on track to produce significantly more physicians-in-training. My own institution’s charter class, for example, embodied the optimism that expanding UME capacity would help alleviate physician shortfalls in our region.

However, this strategy has not translated into a proportional increase in practicing physicians because those graduates still must find residency slots to complete their training. We have, in effect, added more cars to the on-ramp without building new lanes on the highway. The sobering reality is that new medical schools simply feed more graduates into an unyielding bottleneck. National Residency Match statistics illustrate the point. In 2025, over 20% of applicants failed to match [2]. The gap persists. Thanks to the UME expansion, the U.S. now graduates enough new doctors to fill all its residency slots, and then some, but without additional GME positions, many of those doctors cannot complete training. In fact, if one considers just U.S. MD and DO seniors, virtually all of them can currently match into residency. The ones left out are disproportionately international medical graduates and U.S. citizens who studied abroad. But this observation should not breed complacency. Rather, it underlines that even at full capacity, our GME system is barely adequate to absorb the U.S. graduates alone. We rely on international physicians to fill many residency positions, especially in primary care and rural programs, yet still come up short overall. As U.S. medical schools continue to expand enrollment, the pressure on GME will only increase. In the next three years, AAMC projects the number of U.S. med school graduates will grow by another 3,000-4,000 per year, but where will those additional graduates go for residency training [7]?

From my perspective as a former dean, this situation is deeply concerning. We promised students a pathway to practice, but without systemic changes, we are funneling some of them into a dead end. It is telling (and somewhat ironic) that the AAMC itself, having championed medical school growth, now urgently calls on Congress to lift the residency funding caps to avert a shortfall of up to 139,000 physicians by 2033 [6]. The medical education community has recognized that UME expansion alone cannot solve the physician shortage unless GME expands in tandem. Unfortunately, establishing a new medical school is far easier (politically and financially) than expanding residency programs. A new school can be opened with state funding or private investment and will attract tuition-paying students. In contrast, new residency programs require a paying entity, usually a teaching hospital or government, to support resident salaries and training costs, often with no immediate revenue gain. Thus, many states proudly cut ribbons on new medical schools to address physician shortages, yet fail to invest in residency positions for those graduates. The result is a maldistribution of effort and funding. For example, states like Florida and Texas significantly increased medical school seats in the past 15 years, but initially lagged in creating residency positions, leading many homegrown graduates to leave for training elsewhere (and many never return). Opening more schools without bolstering GME is like adding more seeds to a field without enough water or sunlight; the seedlings will wither before they bear fruit.

The current approach is not only ineffective but also inefficient. It usually takes four years and hundreds of thousands of dollars in resources to produce a medical school graduate, who then must sit idle or switch careers if they cannot complete training. Meanwhile, patients in both primary care and specialty areas continue to experience provider shortages. In economic terms, we are underutilizing the human capital we invested in through education. In human terms, we are dashing the hopes of qualified, motivated graduates who want nothing more than to serve patients. This is perhaps most heartbreakingly evident in primary care. Our nation faces a grave shortage of primary care physicians, yet every year, well-trained MDs and DOs who would gladly serve as primary care doctors are not admitted to residency. They are literally physicians without a license to heal. A bottleneck that forces willing physicians to sit on the sidelines is unacceptable in a country facing widespread healthcare access gaps.

Consequences for Patient Care and the Physician Workforce

The residency bottleneck doesn’t just affect medical graduates; it ripples into the care available to every American. By constraining the number of new doctors, the cap contributes to the physician shortages that have become apparent in many fields and regions. The U.S. today has significantly fewer physicians per capita than most other high-income countries. This deficit is not abstract; it translates into real difficulty for patients seeking care. Areas with lower physician-to-population ratios have demonstrably worse health outcomes. For instance, regions with fewer primary care providers have higher mortality rates and lower life expectancy, and communities lacking obstetricians have higher maternal and infant mortality [9]. During my years building systems of care in both urban and rural settings, I saw how a dearth of doctors meant longer wait times, overflowing emergency departments substituting for proper longitudinal care, and physician burnout in those trying to fill the gaps. The COVID-19 pandemic further highlighted the fragility of our physician workforce; some hospitals, especially in rural areas, simply did not have enough in-house doctors to meet surges or to maintain 24/7 coverage, a shortfall that cost lives.

The cap on residency slots also skews which specialties and locations get the limited slots, compounding maldistribution. Medicare’s GME funding formulas historically reward hospitals for residency positions in procedures and specialty care (which generate higher Medicare revenues) more than in primary care or pediatrics, and they favor training in hospital settings over community clinics. Thus, the residency cap not only limits quantity but inadvertently encourages training in subspecialties and urban tertiary centers. Primary care residency programs (family medicine, general internal medicine, etc.) struggle to expand despite societal needs. Likewise, rural hospitals, which often lack large Medicare patient volumes, find it hard to obtain funded residency positions under the current rules. Physicians often practice where they train, so this perpetuates a cycle where rural and underserved areas have fewer residency programs, and subsequently, fewer doctors settle there. In my experience, directing a health system that included rural clinics, we found that establishing a rural residency track was one of the most effective ways to eventually recruit physicians to those communities, but launching such programs required navigating the maze of funding regulations and securing separate, often state-based, support since Medicare funding was maxed out. The BBA’s cap, by tying GME slots to 1996 hospital training levels, effectively ignored population growth and shifts in care needs. Today’s distribution of residency positions bears the imprint of 1990's healthcare utilization patterns, not 2020's realities.

The quality of training can also be impacted. With tight limits on positions, residency programs have become fiercely competitive in many specialties, potentially selecting trainees with extremely high test scores and research credentials at the expense of other important attributes like diversity, community background, or passion for primary care. One could argue that if residency slots were more plentiful, the selection process could prioritize aligning the workforce with need (e.g., choosing more applicants likely to serve in rural primary care) rather than rationing positions based on board exam scores. Moreover, existing residents often face heavier workloads because hospitals cannot hire as many residents as needed, which can impair learning and contribute to burnout. I have seen residency programs where staffing was so lean (relative to patient volume) that residents spent an inordinate amount of time on scut work and administrative tasks, rather than educational activities, a downstream effect of hospitals being unable to fund additional resident positions to spread the work.

Finally, the residency constraint has spurred some controversial stopgap measures. A few states have introduced special licensure pathways to allow unmatched medical graduates to work in limited roles. For example, Missouri created an “assistant physician” license that lets unmatched graduates practice primary care in underserved areas under supervision [10]. While innovative, these measures underscore the desperation of the situation: we are inventing parallel, suboptimal paths rather than fixing the core problem. Similarly, we have leaned more on other healthcare professionals (nurse practitioners, physician assistants) to fill gaps, triggering scope-of-practice debates, rather than ensuring we train enough physicians in the first place. Nurse practitioners and physician assistants are integral to team-based care, but their rise does not obviate the need for more residency-trained physicians, especially for complex cases and leadership roles in care delivery. In sum, the cap on GME has had broad and deep repercussions: stymied careers, underserved communities, overburdened training programs and physicians, and ultimately, patients who struggle to access the care they need.

Toward Solutions: Expanding and Modernizing GME

If the bottleneck in residency training is the root cause of the U.S. physician shortage, then logically, the solution is to expand GME capacity. But simply stating “add more residency slots”, while true, is easier said than done. Training a physician is resource-intensive. Residency slots require funding streams, clinical faculty for supervision, and sufficient patient volume and infrastructure for teaching. The federal cap means that most teaching hospitals have little financial incentive to expand positions beyond what Medicare will support, unless alternative funding is secured. Therefore, solving this issue will require a combination of policy changes, innovative funding, and modernization of training models to use resources more efficiently. Here, I propose and discuss several avenues to expand GME and make it more adaptable to twenty-first-century needs, based on both my leadership experience and emerging ideas in medical education.

Lift or redesign the federal GME cap: The most direct solution is for Congress to act. Numerous bills have been proposed to lift the Medicare residency cap incrementally. For instance, the Resident Physician Shortage Reduction Act has been introduced multiple times (2017, 2019, 2021, 2023), with the latest iteration to add 14,000 Medicare-funded residency slots over 7 years [11]. Thus far, only very modest increases have been enacted. It is imperative that lawmakers recognize GME expansion as a national priority. Investing in residency training yields a direct return in the form of more physicians available to care for an aging population, a clear public good. Some have suggested redirecting unused physician training funds or creating a GME trust fund independent of Medicare, so that training slots are aligned with workforce needs rather than historical Medicare patient loads. Another approach is to mandate that new medical schools be accompanied by new residency positions in that state or region, essentially tying UME growth to GME growth. Federal and state grants could incentivize health systems to create new residency programs in needed specialties (for example, rural family medicine, geriatrics, or psychiatry) by covering startup costs. Lifting the cap, even gradually, is the linchpin; without it, other innovations will have limited reach. As someone who has navigated federal funding regulations, I believe a pragmatic first step is to at least raise the cap in areas of critical shortage (such as primary care, rural hospitals, and Veterans Affairs facilities) where the return on investment in terms of patient access would be greatest. The bottom line is that solving a problem created by federal policy, the 1997 cap, requires federal action. Every year of delay leaves additional thousands of physicians untrained and countless patients untreated.

Public-private partnerships for residency funding: In the absence of sufficient federal support, state and private health systems can and should step up to fund more residency positions. We have started to see examples of this. Some state legislatures have allocated funds to create new residency programs, recognizing that doctors often stay to practice where they train. For example, Texas and Florida in recent years provided state GME funding to expand positions, especially in primary care, to retain their medical school graduates locally. On the private side, large hospital systems and even for-profit entities have gotten into the GME arena. One notable case is HCA Healthcare, a national for-profit hospital chain, which in 2015 began rapidly establishing new residency programs in its hospitals across multiple states. By taking advantage of Medicare’s policy that funds new residency programs for their first five years (even at hospitals that previously had none), HCA managed to grow to become the largest sponsor of residency programs in the U.S., training over 5,000 residents a year in 2020 [12]. This kind of entrepreneurial approach indicates that where there is a will (and some funding leeway), GME can expand even under the cap. Academic medical centers, community hospitals, and consortia can collaborate to share the costs and benefits of training additional residents. For instance, a community hospital might partner with a larger academic center to create a new residency program, with costs split and with the community hospital benefiting from having resident physicians on site, providing care. As a health system executive, I have negotiated such partnerships, and we found that even modest local investments could reap huge dividends in physician supply downstream. The key is aligning incentives so that training a resident is seen not as a financial burden but as an investment in the pipeline of the hospital’s future staff. Loan forgiveness programs could also be structured such that hospitals or states that fund new residency slots receive service commitments from those trainees to work in underserved areas for a number of years after training, further amplifying the return to the public.

Artificial intelligence (AI)-augmented supervision and efficiency: One of the most exciting frontiers for expanding GME capacity is the intelligent use of technology, particularly AI and digital health technologies, to increase the efficiency and reach of clinical training. As someone who has developed digital and neuroscience-based therapeutics, I’ve observed how technology can extend human capabilities. In the context of residency training, AI can be leveraged in several ways to mitigate faculty supervision constraints and enhance learning. For instance, AI-powered decision support systems can help monitor patient care activities and flag potential errors or urgent situations in real time, serving as a safety net when supervising attendings are overseeing larger teams or covering multiple sites. In essence, physicians could “supervise AI-augmented care systems” rather than personally performing every routine task. This concept means one attending physician might safely mentor more residents if some of the cognitive load (e.g., tracking patients’ data trends, protocol adherence, documentation) is offloaded to AI tools. Such systems are already emerging, and AI algorithms can scan for deviations in care protocols or alert attendings to a resident’s difficult case that might need input. Additionally, AI can streamline documentation (e.g., AI scribes generating draft notes), reducing the scut work for trainees and allowing them to focus on learning and direct patient care. By automating administrative tasks and routine surveillance, AI frees up both trainees and faculty to handle a larger volume of educational cases without compromising quality.

Furthermore, AI and machine learning can personalize medical education. Adaptive learning platforms can identify a resident’s weaknesses (i.e., in interpreting ECGs, central line placement, or following local antibiotic guidelines) and push targeted educational content or simulations to address those gaps. We are already seeing AI tutors in surgical training: studies have shown that an AI-based virtual tutor can effectively teach surgical skills, in some cases outperforming human instructors in improving trainee performance. Imagine each resident having a virtual coach that provides immediate feedback on their diagnostic plans or operative technique, supplementing the teaching from human attendings. This could accelerate competency acquisition, potentially shortening the time needed to train or allowing residents to progress faster through certain rotations. In continuing medical education and faculty development, augmented intelligence could also train attending physicians to supervise more effectively and efficiently. Importantly, none of this means replacing physician teachers or reducing standards; rather, it means extending expert supervision through technology and making training more output-focused. With AI assistance, the limiting factor for residency expansion (the number of patients a given attending can oversee and the time for teaching) can be relaxed. The ACGME and accrediting bodies are beginning to explore these possibilities, and pilot programs for virtual supervision (for example, attending physicians overseeing residents via tele-video for certain encounters) have shown that it can be done without loss of quality. In fact, during the COVID-19 pandemic, Medicare temporarily allowed real-time audiovisual supervision of residents and found it to be effective; making such flexibility permanent could enable residency programs to utilize attendings from anywhere in the country to help supervise trainees remotely [13]. Embracing these technological aids can allow us to safely increase the ratio of trainees to faculty and spread teaching capacity across geographies, which in turn allows more residents to be trained.

Hybrid and distributed training models: Another promising avenue is to break free from the traditional “one hospital, one program” model of GME in favor of more distributed and flexible training pathways. The rigidity of needing a large academic medical center to serve as the training hub is fading. We are seeing growth in consortium-based residencies where a network of hospitals and clinics collaboratively train a cohort of residents, sharing resources. For example, a resident might rotate through a university hospital for specialty rotations but spend significant time at community hospitals or outpatient clinics that serve as additional training sites. This not only increases capacity (since multiple smaller centers can host residents) but also trains physicians in the settings where we need them most (community and rural practice). Rural Training Tracks (RTTs) are a proven model in primary care: a residency program can base first-year training in a tertiary hospital and the later years in a rural hospital or clinic, with Medicare providing special support for RTT positions [14]. Expanding such models can directly pipeline doctors into rural areas. In my time, coordinating a multi-hospital health system, we implemented a hybrid training program in psychiatry that connected a major academic medical center with outlying mental health clinics via tele-mentoring. Residents would see patients in person at remote clinics but join case conferences and didactics via video, with faculty traveling periodically. This allowed us to increase the psychiatry residency class size and deliver services to underserved areas simultaneously. The success of that pilot suggests that many smaller or underserved communities could “host” residents if we leverage telecommunication and regional partnerships for supervision and education.

Additionally, shortened or accelerated training pathways can help. The traditional residency lengths (3 years for primary care or internal medicine, 4+ years for most specialties) are somewhat arbitrary and based on time served rather than competency. There is growing interest in competency-based medical education (CBME) where residents progress upon mastering skills, not just after a set number of years [15]. A highly competent resident could finish sooner, freeing up a slot for another trainee. Some specialties and programs (e.g., pilots in family medicine and internal medicine) have experimented with CBME and early graduation for those who achieve proficiency, and while challenges remain, it is an idea worth expanding. Likewise, combined degree programs (such as 3-year MD programs that feed directly into a residency at the same institution) can smooth the continuum and ensure that those students have a residency slot secured. Several U.S. medical schools now offer a condensed 3-year MD with a guaranteed residency position in primary care or selected fields at their affiliated hospitals. These programs not only shorten training by a year but also eliminate the uncertainty of the Match for those students, effectively aligning UME and GME for a subset of trainees. If scaled up, such approaches could modestly increase the output of practicing physicians without needing a proportional increase in residency spots (because the training duration is shorter for some).

Learning from international models: Many peer countries manage the transition from medical school to practice in a more coordinated manner, and while healthcare systems differ, we can draw lessons from them. In the United Kingdom, for instance, virtually all medical school graduates are guaranteed a slot in a two-year Foundation Programme (equivalent to a rotating internship/residency) as part of the National Health Service’s workforce planning [16]. The UK deliberately calibrates the number of medical school entrants to roughly match postgraduate training posts for its grads (occasionally with slight shortfalls, but nothing like the scale seen in the U.S.). Canada similarly went through a period of cutting medical school and residency numbers in the 1990s (much like the U.S.), only to face shortages; they reversed course and now closely coordinate UME and GME output, though they still struggle with rural physician supply [17]. The lesson here is the importance of national workforce planning: treat the pipeline as a whole. The U.S. lacks any centralized mechanism to align the number of medical school seats, residency positions, and societal needs. Medicare funding was a de facto control, but one divorced from actual workforce requirements. Perhaps it is time for a national commission or task force to formally project physician supply and demand by specialty and region, and then empower funding bodies (Medicare, Medicaid, VA, etc.) to target GME growth accordingly. Other countries also show the value of community-based training. In Australia, for example, the government expanded rural clinical schools and regional residency programs to address physician maldistribution, with promising results in retaining doctors in those areas [18]. Many European nations require return-of-service commitments (e.g., serving in a needed area) in exchange for subsidized training slots, an approach that could be considered for any new U.S. federally funded GME positions to ensure they alleviate shortages in underserved communities [19].

Finally, there are IMGs already trained elsewhere who could help alleviate U.S. shortfalls if we modernize pathways for their integration. While the solution to the U.S. shortage should primarily be training more U.S. physicians, selectively leveraging global talent is wise. Streamlined certification programs (such as allowing experienced foreign doctors expedited entry into U.S. residency or practice under supervision) could add physician capacity in the short run, as some experts have suggested. Some states have looked at easing licensure for IMGs in certain specialties or granting them provisional licenses. Such measures, while sensitive and needing careful quality control, recognize that the U.S. does not operate in isolation; our residency bottleneck is also a barrier to qualified international physicians who could serve American patients. Internationally trained physicians have long filled gaps in U.S. healthcare - about one in four doctors practicing in the U.S. is an IMG. Facilitating their contribution while we build domestic training capacity is a win-win.

From my vantage point as an educator, clinician, and innovator, the diagnosis of America’s physician shortage is clear: we have a pipeline problem, not at the entry point of medical school, but at the critical junction of residency training. We have bright, altruistic students in ample supply and our medical schools are graduating more physicians than ever before, yet a decades-old decision to cap residency slots has left those new doctors without the necessary pathway to practice. The result is a generation of would-be healers forced to stand idle, even as patients languish on waiting lists and communities struggle to recruit physicians. Addressing this issue requires leadership and investment to expand GME, the kind of bold, structural change that does not fit neatly in a soundbite but will pay dividends in health for decades to come. We must advocate for lifting the arbitrary cap of 1997 and funding enough residency positions to meet our actual healthcare needs. We must realign incentives so that training a doctor is viewed as a societal investment, not a hospital expense to be minimized. Further, we should embrace innovation, deploying technology, redesigning curricula, and learning from global best practices, to make our training system more efficient and equitable.

I have dedicated my career to advancing medical education and patient care, from building new academic programs to pioneering digital health solutions. These experiences leave me optimistic that we can rise to this challenge. The same spirit of innovation that drives therapeutic breakthroughs can be applied to how we train the next generation of physicians. Imagine a future in which residency positions are plentiful enough that no medical graduate goes unmatched; where intelligent systems support attending physicians so they can supervise larger teams without compromising quality; and where a young doctor’s ability to serve isn’t limited by the year her hospital’s Medicare cap was set. That future is within reach if we summon the will to change. The physician shortage is not an unsolvable enigma; it is a human-made bottleneck, and we can open it.

Conclusion

The physician shortage in the United States is not a matter of insufficient medical school graduates, but of a residency bottleneck entrenched by outdated policy. The 1997 Medicare cap on residency slots remains the most significant barrier to expanding our physician workforce [1]. While medical schools have proliferated, graduates are increasingly stranded without a path to licensure. To resolve this, we must invest in graduate medical education by lifting federal caps, incentivizing public-private partnerships, and modernizing training with technological and hybrid innovations. Without a robust and scalable residency system, we will continue to waste human capital and leave patient needs unmet. The fix is not more medical schools, but more places to finish training the doctors we already have.
